# Effects of Harvest Maturity on the Fruit Quality of Different Flesh-Type Peach Stored at Near-Freezing Point Temperature

**DOI:** 10.3390/foods11152200

**Published:** 2022-07-24

**Authors:** Binbin Zhang, Xingxing Chen, Na Wang, Shaolei Guo, Weibing Jiang, Mingliang Yu, Ruijuan Ma

**Affiliations:** 1 Institute of Pomology, Jiangsu Academy of Agricultural Sciences/Jiangsu Key Laboratory for Horticultural Crop Genetic Improvement, Nanjing 210014, China; binbin@jaas.ac.cn (B.Z.); guoshaolei0305@126.com (S.G.); mly1008@aliyun.com (M.Y.); 2 College of Horticulture, Nanjing Agricultural University, Nanjing 210095, China; 2017104018@njau.edu.cn (X.C.); nawang0622@163.com (N.W.); weibingj@sohu.com (W.J.)

**Keywords:** *Prunus persica*, external quality, internal quality, cold storage, maturity

## Abstract

To investigate the peach fruit flesh types (soft-melting, hard-melting, stonyhard and non-melting) and harvest maturity level suitable for near-freezing temperature storage (NFTS), eight peach cultivars that had four flesh types were used as test materials. Changes in fruit respiration intensity and ethylene release rates, as well as the differences in quality indexes, such as soluble solids content (*SSC*), firmness, color difference, pigment content, soluble sugar and organic acid component content, of three fruit maturity levels (70%, 80% and 90% maturity) under NFTS conditions were analyzed and compared. The fruit quality indexes of peach having different maturity levels and flesh types changed little during NFTS. The *SSC* and total sugar content of hard-melting and stonyhard peach fruit were higher than those of other flesh types during NFTS. Those fruit maintained greater firmness at the end of the storage period. The differences in respiration intensity and ethylene release rate were small, but for fruit coloring, hard-melting fruit performed better than stonyhard fruit. The 80%, compared with the 90%, maturity stage maintained more fruit moisture, had less fruit mass loss and maintained a greater edible firmness. It effectively impeded the fruit senescence process and was the most suitable maturity for NFTS. Thus, the hard-melting peach maintained the highest commercial value and desirable storage characteristics under NFTS conditions, and its 80% maturity level was the most suitable for NFTS.

## 1. Introduction

Peach [*Prunus persica* (L.) Batsch], which originated in China, is considered to be a delicious and healthy summer fruit in most temperate regions of the world. The planting area and production of peach in China were 7.78 × 10^5^ ha and 1.5 × 10^7^ t in 2020 according to the data of the Food and Agriculture Organization (FAO), which took 52.14% and 61.05% of the world’s planting area and production, respectively. However, peach is a typical climacteric fruit that easily softens and rots quickly after harvesting. Thus, it has a short shelf life, which is not conducive to storage and transportation [1,2]. Generally, peach needs to be refrigerated after harvesting, but fruit suffer from chilling and freezing injuries when refrigerated at low temperatures [3]. The fruit flesh texture becomes poor, loses flavor, fails to post-ripen and soften normally, and even undergoes a series of physiological changes, such as flesh browning [4,5], resulting in poor storage effects in a short storage time. This decreases the edible and commercial values of fruit after storage.

There are many factors that affect fruit preservation such as the role of the cultivars and their general characteristics besides fruit flesh-melting. In addition, proper storage temperature and harvest maturity are very important. Peach is a cold-sensitive fruit, and cold or freezing damage phenomena displayed during low temperature storage are dependent on the harvest maturity of the fruit. The sensitivity of fruit with different maturity levels to low temperature differ. The greater the maturity, the less the sensitivity to low temperature [6]. Shewfelt et al. [7] studied the effects of different harvest maturity levels on fruit physiological quality during low-temperature storage and found that peach cultivars (cv. ‘Loring’ and ‘Redglobe’) stored in a 5 °C environment soften faster with increasing maturity level, and their commercial and edible values are greatly reduced. Therefore, the harvest maturity level has a great influence on the storage effects on peach fruit. Appropriate harvest maturity is an important guarantee for maintaining the fruit commodity value and extending the shelf life. The impacts of harvest maturity on storage and transportation quality of a cultivar of fruit, including carambola [8], plum [9], loquat [10], mango [11] and banana [12], have been studied. In recent years, the effects of harvest maturity on the quality of peach fruit have also been studied, but most focused on normal and low-storage temperatures greater than 0 °C. No systematic study of the quality changes of peach having different flesh types under near-freezing point temperature storage (NFTS) has been performed. Near freezing point temperature (NFT) is the storage temperature between the super-cooling point and freezing point of an individual material, which is within the minimal non-frozen temperature range [13].

The aim of this work was to systematically observe, analyze and compare quality indexes of peach fruit with different flesh types and maturity (70%, 80% and 90% maturity) levels under NFTS conditions. In addition, changes in fruit respiration metabolism and ethylene release rate were also determined. The results help to further clarify the peach fruit flesh type suitable for NFTS, and they provide a theoretical basis for determining the harvest maturity level suitable for NFTS.

## 2. Materials and Methods

### 2.1. Plant Material and Experimental Design

The experiments were carried out in the Peach Experimental Park of Jiangsu Academy of Agricultural Sciences (32°2’ N, 118°52’ E, altitude 11 m) in 2018. Eight 7-year-old peach cultivars having the four flesh types, namely soft-melting (‘Yuhualu’, ‘Xiahui 5’), hard-melting (‘Xiahui 6’, ‘Hujingmilu’), stonyhard (‘Xiacui’, ‘Qinwang’) and non-melting (‘Frederick’, ‘Babygold 9’) were used as the experimental materials using ‘Maotao’ as rootstocks. Six healthy trees of each cultivar were grown in the same manner. The trees each had three main branches, and were planted with a row spacing of 3 m × 5 m. They were cultivated in the north-south direction with ridging, and they were managed in accordance with conventional cultivation measures.

At the appropriate maturity time for each cultivar, fruit subjected to good light conditions from the periphery above the middle of the crown were harvested in batches, and then they were quickly transported to the laboratory. Fruit of uniform size and free from diseases and insect pests were selected randomly for each maturity level. There was no continuously rainy weather for 10 days before harvest, and this had no obvious effects on fruit quality.

The selected peach fruit were placed in shallow foam boxes (single layer) on the basis of the three maturity levels (70%, 80% and 90% maturity) and covered with PE plastic wrap. Fruit maturity was determined according to both harvest experience (the base color of the peel) and the index of absorbance difference (*I*_AD_), which measures the loss of chlorophyll as fruit ripen by comparing absorbance levels at 670 and 720 nm [14]. The *I*_AD_ on the middle of the two sides of the fruits of each cultivar was measured by a DA-Meter (TR Turoni srl, Forlì, Italy). The grading standards for the fruit maturity of different peach cultivars are shown in Table 1. The external temperature controller (ZDR-1000P, Hangzhou Zeda Instrument Co., Ltd., Hangzhou, China) was used to set the conditions of the freezer for the near-freezing point temperature and dark storage test. Samples were observed every 4 days at near-freezing point temperature, and 15 fruit were sampled per time for the determination of relevant indicators, with five fruits serving as biological replicates. From each peeled fruit, the peel and adaxial flesh on both sides of the suture was collected, minced, homogenized, frozen in liquid nitrogen and stored at −80 °C until analysis.

### 2.2. Measurements

#### 2.2.1. Freezing Point Temperature of Peach Fruit

The freezing point temperature of peach fruit was measured using a ZJ10X series multi-channel high-precision temperature tester (Changzhou Zhongjie Electronics Co., Ltd., Changzhou, China). The probe of the recorder was inserted vertically between the pericarp and the core of the fruit, and the whole fruit was placed into a low-temperature freezer at −20 °C. The fruit was placed in the center of the freezer to avoid contact with the freezer walls. The door of the freezer was closed tightly. The pulp temperature was automatically recorded every 15 s, and the recording period lasted for more than 12 h. Then, the data were exported, and the temperature change curves were constructed to determine the freezing point temperature.

#### 2.2.2. Mass Loss Rate

In total, 10 fruit per maturity level of each cultivar were labeled in sequence, counted and weighed. They were then immediately returned to the original storage environment to calculate the mass loss rate of the fruit.

#### 2.2.3. Respiration Rate

A gas analyzer (Check Mate 3, PBI Dansensor, Ringsted, Denmark) was used to measure the respiration rate of peach fruit, and this was expressed as the amount of CO_2_ released per unit mass at 25 °C per unit time. After weighing the fruit and recording the mass (*m*), it was placed in a 4.5-L airtight container at 25 °C for 1 h. Then, the instrument was connected to measure the CO_2_ concentration (*C*), and finally, the volume (*V*) of each group of fruit was determined using the improved drainage method. The calculation formula was as follows:

Respiration rate (mg kg^−1^ h^−1^) = 10^3^ × (4.5 − *V*) × *C* × 1.96/*m*

#### 2.2.4. Firmness and Ethylene Release Rate

The fruit flesh texture was measured using a TA-XT Plus texture analyzer (Stable Micro-Systems Texture Technologies Co., Scarsdale, NY, USA) fitted with an 8-mm-diameter probe. Firmness values were expressed in Newtons (N). Firmness studies were conducted at a pre-test probe rate of 1.0 mm s^−1^ and a distance of 5 mm. The ethylene release rate of peach fruit was expressed using the ethylene release per unit fresh weight per unit time. Each peach fruit was placed in a closed container, and the gas was extracted to determine the ethylene release after 2 h. The instrument used for ethylene determination was a GC-7890A gas chromatograph (Agilent). The chromatographic conditions were as followed: FID detector, Hp-Plot q capillary column (20 m × 0.53 mm × 20 μm), split ratio of 10, He loaded, 40 °C column temperature, 220 °C detector temperature, and 1 mL injection volume. The characteristics were measured using fresh fruit and repeated three times.

#### 2.2.5. Peel Color Difference and Pigment Measurements

The red saturation (a*) and yellow saturation (b*) of the peel were measured using a Color Quest XE color difference meter (Hunter Lab, Reston, VA, USA) on both sides of the abdomen of the fruit suture, and the ratio of red-green difference/yellow-blue difference was calculated (a*/b*). The average of two points was used as the corresponding index value of the fruit. The content of chlorophyll (Chl) in the pericarp was determined using the method of Lichtenthaler and Wellburn [15], followed by extraction with 95% ethanol and measuring the absorbance at 665 and 649 nm. The anthocyanin (Ant) content was determined in accordance with the method of Zapsalis and Francis [16], followed by extraction with 1% methanol solution of hydrochloric acid, and measuring the absorbance values at 650, 620 and 530 nm. Then, the content was calculated.

#### 2.2.6. Soluble Solids Content, Soluble Sugar and Organic Acid Content

The juice of the pulp near the middle of the suture of each fruit was sampled, and the soluble solids content (*SSC*) was determined using a PAL-1 refractometer (ATAGO. Itabashi-ku, Japan). The average of two points was used as the *SSC* of each fruit. The contents of soluble sugars (sucrose, glucose, fructose and sorbitol) and organic acids (malic acid, quinic acid and citric acid) in the pulp were determined using Agilent 1100 high-performance liquid chromatography (Agilent Technology, Santa Clara, CA, USA) [17]. The total sugar and acid contents equaled the total content of each soluble sugar and acid component, respectively. The sugar-acid ratio was calculated using the total sugar and total acid contents.

#### 2.2.7. Acceptability

Acceptability of 8 peach cultivars with different maturity degrees after NFTS for 16 days was determined through a consumer test. The evaluation guideline for acceptability used a hedonic scale marked with two anchors: 0 = dislike extremely; and 15 = like extremely [18].

### 2.3. Statistical Analyses

Significant differences between the average values obtained for fruit maturity levels (*I*_AD_ value) of different peach cultivars were evaluated using analysis of variance techniques (ANOVA) and Multiple Ranges Duncan’s test was used to differentiate between the means (*p* < 0.05) using SPSS software, version 23.0 (IBM, Armonk, NY, USA) (Table 1). The same method of ANOVA was also conducted on the analysis of “acceptability”. Microsoft Excel 2018 software was used for statistical analyses and constructing graphics.

## 3. Results

### 3.1. Freezing Point Temperature of Peach Fruits with Different Fleshy Types and Maturity

The freezing point temperatures of peach fruit with different flesh types at different maturity stages are shown in Table 2. The freezing point temperatures of the 90% maturity fruit of soft-melting cultivars were higher than both the 70% and 80% maturity fruit, which had positive values. Neither stonyhard nor non-melting peach cultivars had lower freezing point temperatures at different maturities, and they had negative values. For the hard-melting peach cultivars, the freezing point temperatures were close to 0 (positive value, ‘Xiahui 6’) or below 0 (negative value, ‘Hujingmilu’).

### 3.2. Change in the Mass Loss Rate under Near-Freezing Point Temperature Storage

**Figure 1:**
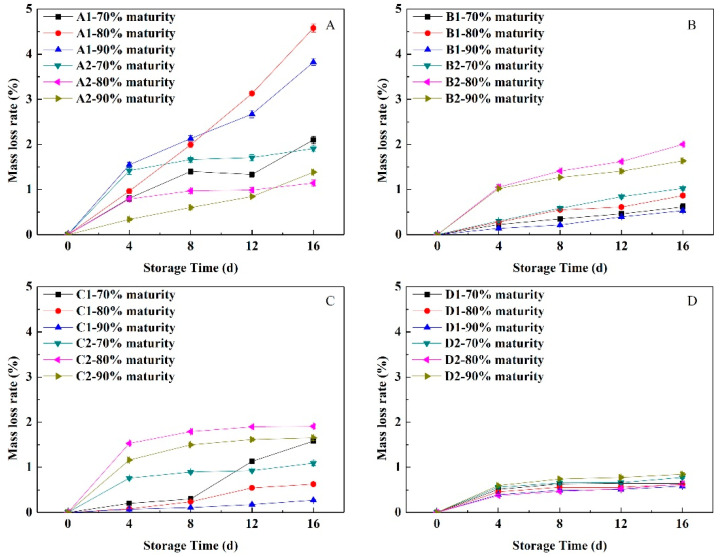
Changes in the mass loss rates of peach fruit having different flesh types during near-freezing temperature storage (NFTS). A1, A2 represent ‘Yuhualu’, ‘Xiahui 5’; B1, B2 represent ‘Xiahui 6’, ‘Hujingmilu’; C1, C2 represent ‘Xiacui’, ‘Qinwang’; D1, D2 represent ‘frederic’, ‘Babygold 9’. Soft-melting (**A**), hard-melting (**B**), stonyhard (**C**), non-melting (**D**). Each point represents means ± SE of three replicates.

As shown in Figure 1, with the extension of NFTS time, the mass loss rates of peach fruit with different fleshy types and maturity levels showed upward trends, but the rates of fruit water loss were quite different. Compared with other fleshy peach cultivars, the water loss rates of the soft-melting peach were the fastest. The mass loss rates of hard-melting ‘Xiahui 6’ and ‘Hujingmilu’, ranked from high to low, were 80%, 90% and 70% maturity. The mass loss rate of the hard-melting fruit was less than 2%. The mass loss rates of hard-melting, stonyhard and non-melting fruit increased fastest in the first 4 days, and then, they increased gradually, which indicated that the water loss rates of the fruit were the fastest in the early stages of NFTS. The non-melting fruit had the lowest mass loss (no more than 1%), and the difference was small, indicating these peach fruits retain moisture under NFTS conditions. In addition, fruit wilting was not observed during NFTS.

### 3.3. Change of Firmness under Near-Freezing Point Temperature Storage

**Figure 2:**
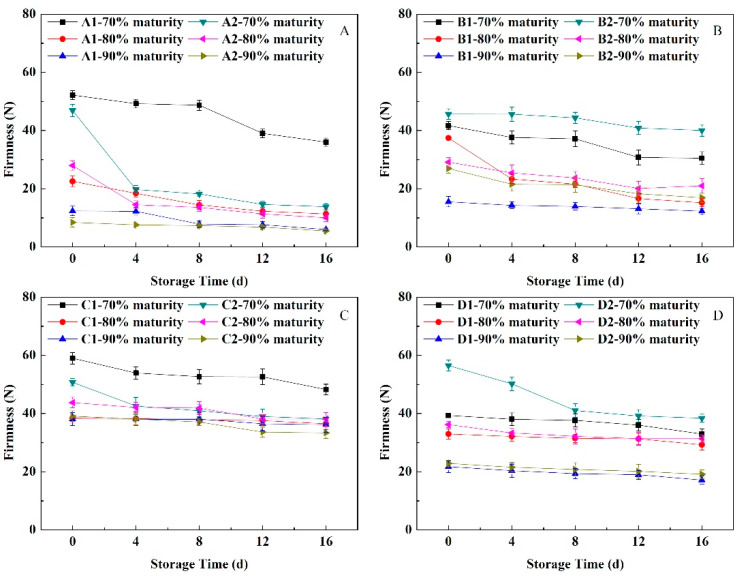
Changes in the firmness of peach fruit having different flesh types during NFTS. A1, A2 represent ‘Yuhualu’, ‘Xiahui 5’; B1, B2 represent ‘Xiahui 6’, ‘Hujingmilu’; C1, C2 represent ‘Xiacui’, ‘Qinwang’; D1, D2 represent ‘frederic’, ‘Babygold 9’. Soft-melting (**A**), hard-melting (**B**), stonyhard (**C**), non-melting (**D**). Each point represents means ± SE of three replicates.

As shown in Figure 2, the firmness of peach fruit with different flesh types and maturity levels showed a downward trend but had different rates of decline during NFTS, with the rate of the soft-melting fruit decreasing fastest. The firmness of three maturity levels of ‘Yuhualu’ fruit decreased gently as the NFTS time increased. ‘Xiahui 5’ had a slower decline in firmness at the 80% maturity level than at the 70% level. The 70% maturity hard-melting fruit had a slower decline in hardness than the soft-melting fruit, and the firmness change trends of the 80% and 90% fruit were similar to those of the soft-melting fruit. The firmness of the three maturity levels of stonyhard ‘Xiacui’ and ‘Qinwang’ fruit showed slow downward trends, and there were no obvious changes at 90% maturity. The firmness of the three maturity fruits of ‘Frederick’ did not change significantly with increased storage times. The firmness of the 70% maturity fruit was greater than those of the 80% and 90% maturity fruit at the end of NFTS, but the firmness of the 80% and 90% maturity fruit decreased slowly. Thus, the higher the fruit maturity level, the better the firmness of the fruit under NFTS.

### 3.4. Change in Peel Color under Near-Freezing Point Temperature Storage

**Figure 3:**
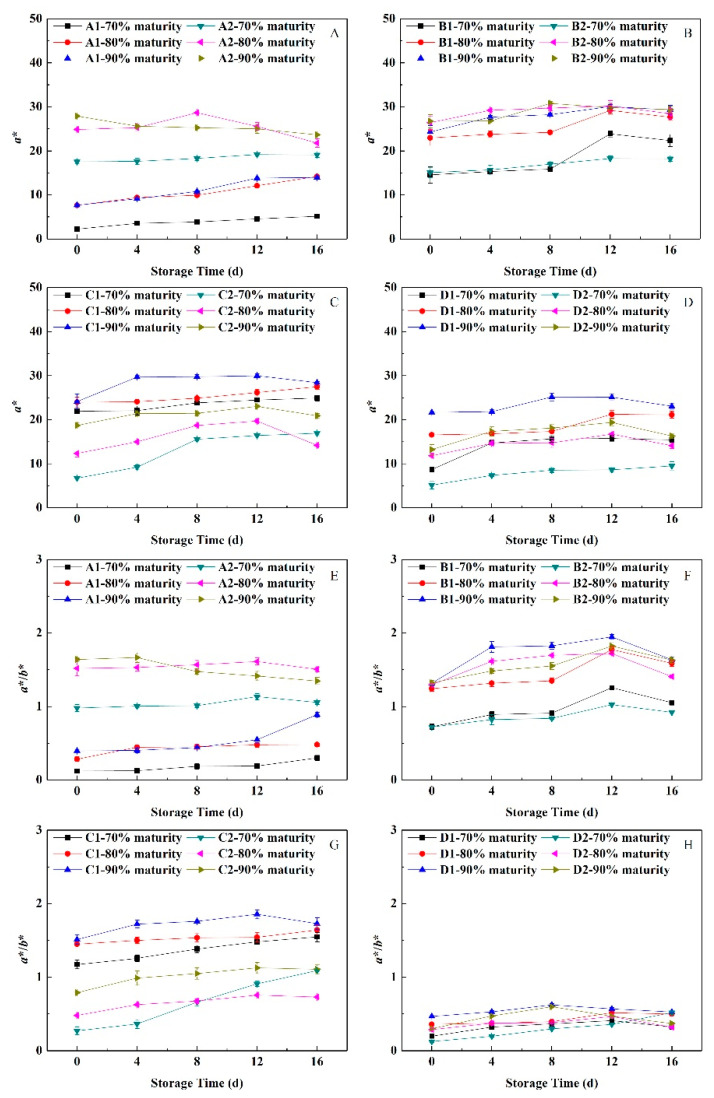
Changes in the chromatic aberrations of peach peel having different flesh types during NFTS. A1, A2 represent ‘Yuhualu’, ‘Xiahui 5’; B1, B2 represent ‘Xiahui 6’, ‘Hujingmilu’; C1, C2 represent ‘Xiacui’, ‘Qinwang’; D1, D2 represent ‘frederic’, ‘Babygold 9’. Soft-melting (**A**,**E**), hard-melting (**B**,**F**), stonyhard (**C**,**G**), non-melting (**D**,**H**). Each point represents means ± SE of three replicates.

The value of a*/b* can indicate the color of fruit during storage. As shown in Figure 3, the a* and a*/b* values of peels from peach fruit having different flesh types before storage were greater at 90%, 80% and 70% maturity, successively. During the NFTS, the a* and a*/b* values of peels from hard-melting peach were the highest overall, and the lowest were the peels from non-melting peach. The a* and a*/b* values of peach peels at different maturity levels increased slowly during the early stages of NFTS. Thus, the red surface color of the fruit deepened continuously. Additionally, there was a downward trend in the later storage period, but the change was small, indicating that NFTS could maintain the peel’s appearance and color.

### 3.5. Change in Peel Pigment Content

#### 3.5.1. Change in Chlorophyll Content

**Figure 4:**
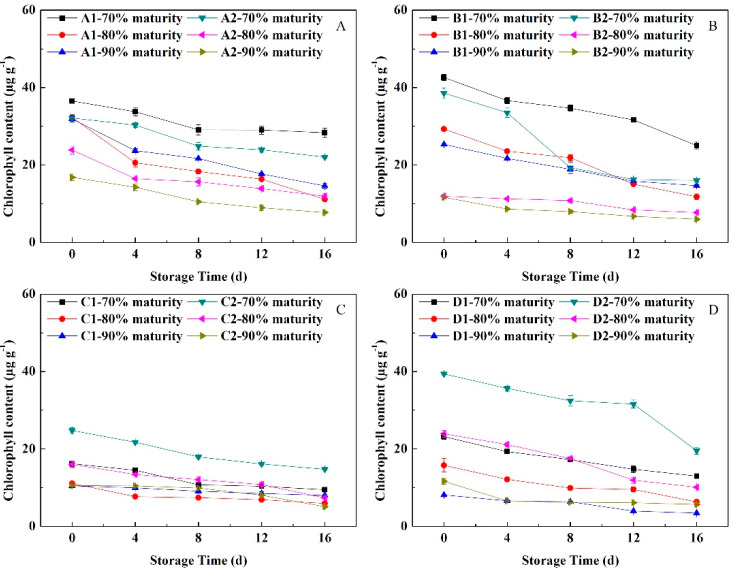
Changes in the chlorophyll contents of peach peel having different flesh types during NFTS. A1, A2 represent ‘Yuhualu’, ‘Xiahui 5’; B1, B2 represent ‘Xiahui 6’, ‘Hujingmilu’; C1, C2 represent ‘Xiacui’, ‘Qinwang’; D1, D2 represent ‘frederic’, ‘Babygold 9’. Soft-melting (**A**), hard-melting (**B**), stonyhard (**C**), non-melting (**D**). Each point represents means ± SE of three replicates.

With the extension of the NFTS time, the Chl contents of peach peel having different flesh types and maturity levels showed downward trends. As shown in Figure 4, the Chl contents in the peels on the day of harvest increased in the following order: 70%, 80% and 90% maturity. The Chl level of the peels at 90% maturity of soft-melting ‘Yuhualu’ and ‘Xiahui 5’ declined faster than that of the peels at 80% maturity. The Chl content in the peels of ‘Xiahui 6’ and ‘Hujingmilu’ at 70% maturity showed a nearly linear downward trend. Those at 80% and 90% maturity decreased slowly, and the level in ‘Hujingmilu’ decreased less than that of ‘Xiahui 6’. The same was true for hard-melting peach. The Chl content in the peel of non-melting peach showed a tendency to decrease slowly and uniformly during NFTS.

#### 3.5.2. Change of Anthocyanin Content

**Figure 5:**
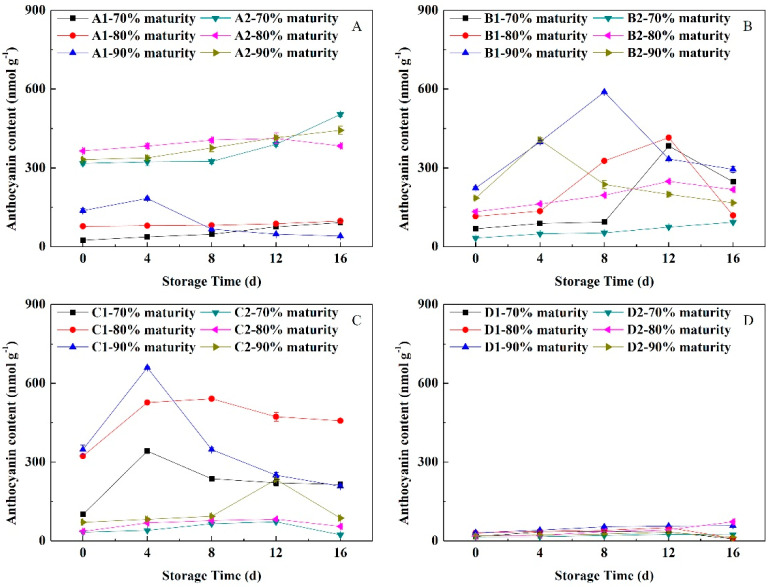
Changes in the anthocyanin contents of peach peel having different flesh types during NFTS. A1, A2 represent ‘Yuhualu’, ‘Xiahui 5’; B1, B2 represent ‘Xiahui 6’, ‘Hujingmilu’; C1, C2 represent ‘Xiacui’, ‘Qinwang’; D1, D2 represent ‘frederic’, ‘Babygold 9’. Soft-melting (**A**), hard-melting (**B**), stonyhard (**C**), non-melting (**D**). Each point represents means ± SE of three replicates.

The change trend of the Ant content in the peels of peach having different maturity levels during NFTS was opposite that of the Chl content. As shown in Figure 5, the Ant content of soft-melting ‘Yuhualu’ at 80% maturity showed an upward trend, but it increased more slowly than at the 70% maturity. The Ant content of ‘Xiahui 5’ at 70% maturity increased rapidly during 8 days prior to storage and continued to increase rapidly. The content in fruit at 80% maturity increased more slowly than that at 90% maturity. The Ant content of ‘Xiahui 6’ peels increased slowly during the early storage period and then increased rapidly after peaking on the 12th day of storage. The Ant content in fruit peel at 90% maturity increased faster than those at 70% and 80% maturity. The content of Ant in ‘Xiacui’ peel with stonyhard gradually decreased after reaching the peak on the 4th day of storage. The Ant content in the peel of the three maturities of ‘Qinwang’ differed slightly, and all reached the peak when stored for 12 days. The Ant content in peach with non-melting increased with the storage time prolonged and the content was the lowest. In summary, the fruit had pigment metabolism under NFTS conditions and the higher the maturity, the faster the Ant decomposition rate in the peel.

### 3.6. Change of Respiration Rate

**Figure 6:**
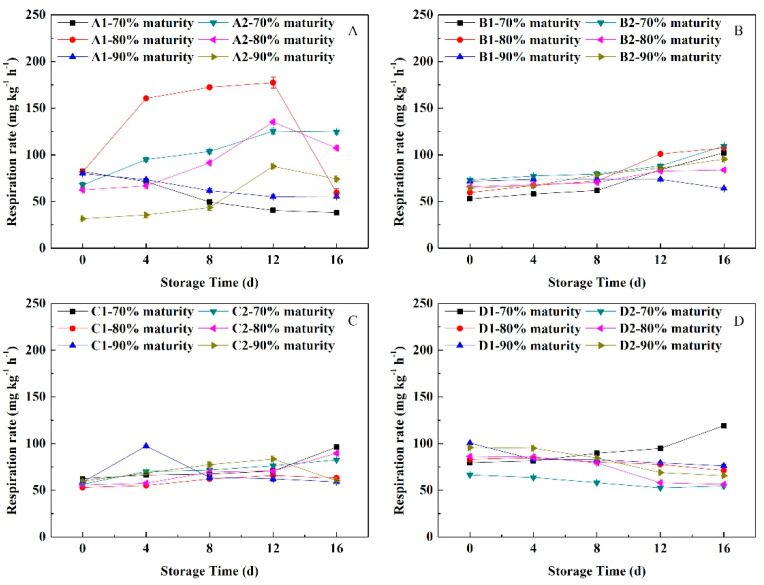
Changes in the respiration rates of peach fruit having different flesh types during NFTS. A1, A2 represent ‘Yuhualu’, ‘Xiahui 5’; B1, B2 represent ‘Xiahui 6’, ‘Hujingmilu’; C1, C2 represent ‘Xiacui’, ‘Qinwang’; D1, D2 represent ‘frederic’, ‘Babygold 9’. Soft-melting (**A**), hard-melting (**B**), stonyhard (**C**), non-melting (**D**). Each point represents means ± SE of three replicates.

As shown in Figure 6, the respiration rates of peach fruit having different maturity levels and flesh types differed. With the extension of NFTS time, the respiration rate of ripened ‘Yuhualu’ fruit at 80% maturity increased rapidly and then decreased, whereas those at 70% and 90% maturity decreased gradually. The respiration rate of ‘Xiahui 5’ increased slowly and showed a downward trend after 12 days of storage. The respiration rates of ‘Xiahui 6’ at 70% and 80% maturity levels increased gradually during storage, whereas that at 90% maturity remained stable and began to decrease on the 12th day of storage. The respiration rates of ‘Hujingmilu’ at all the three maturity rates increased slowly. The respiration rate of ‘Xiacui’ at 90% maturity decreased slowly after peaking 12 days earlier at 70% maturity. The respiration rate of ‘Qinwang’ at 70% and 80% maturity levels increased slowly with the prolongation of NFTS time, and the rate at 90% maturity increased to a maximum on the 12th day of storage. The respiration rate of non-melting ‘Frederick’ at 70% maturity gradually increased during storage, whereas that of ‘Babygold 9’ at all three maturity levels gradually decreased. Respiration rate is an important indicator for evaluating fruit metabolism. The respiration rate of the fruit showed a slow increasing or decreasing trend, indicating that the respiration-related metabolic activity of the fruit decreased with the extension of the NFTS time.

### 3.7. Change in Ethylene Release Rate

**Figure 7:**
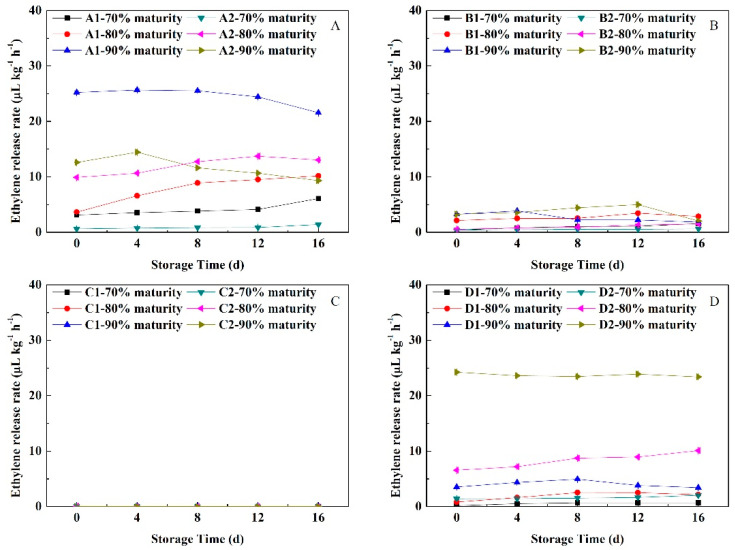
Changes in the ethylene production rates of peach fruit having different flesh types during NFTS. A1, A2 represent ‘Yuhualu’, ‘Xiahui 5’; B1, B2 represent ‘Xiahui 6’, ‘Hujingmilu’; C1, C2 represent ‘Xiacui’, ‘Qinwang’; D1, D2 represent ‘frederic’, ‘Babygold 9’. Soft-melting (**A**), hard-melting (**B**), stonyhard (**C**), non-melting (**D**). Each point represents means ± SE of three replicates.

As shown in Figure 7, during NFTS, the ethylene release rates of different fruit flesh was soft-melting > non-melting > hard-melting > stonyhard. With the extension of NFTS time, the release rates of ethylene from Yuhualu’ and ‘Xiahui 5’ at the 70% and 80% maturity levels ‘increased slowly. The ethylene release rate of ‘Yuhualu’ at 90% maturity showed a downward trend after being stable for 8 days before storage, whereas that of ‘Xiahui 5’ gradually decreased after peaking in the first 4 days. The release rates of ethylene from ‘Xiahui 6’ and ‘Hujingmilu’ at 90% maturity peaked on the 4th and 12th days of storage, respectively, whereas those at 70% and 80% maturity gradually increased with the extension of NFTS time. During the storage period, the ethylene release rate of non-melting peach showed the smallest change, increasing and decreasing slowly at 70%, 80% and 90% maturity. The stonyhard peach fruit with had a small amount of ethylene released on the day of harvest, but ethylene was almost undetectable during NFTS, indicating that the fruit did not produce much ethylene when stored at a near-freezing point temperature.

### 3.8. Changes in Soluble Solids Content, Soluble Sugar Content and Sugar-Acid Ratio

#### 3.8.1. Change in Soluble Solids Content

**Figure 8:**
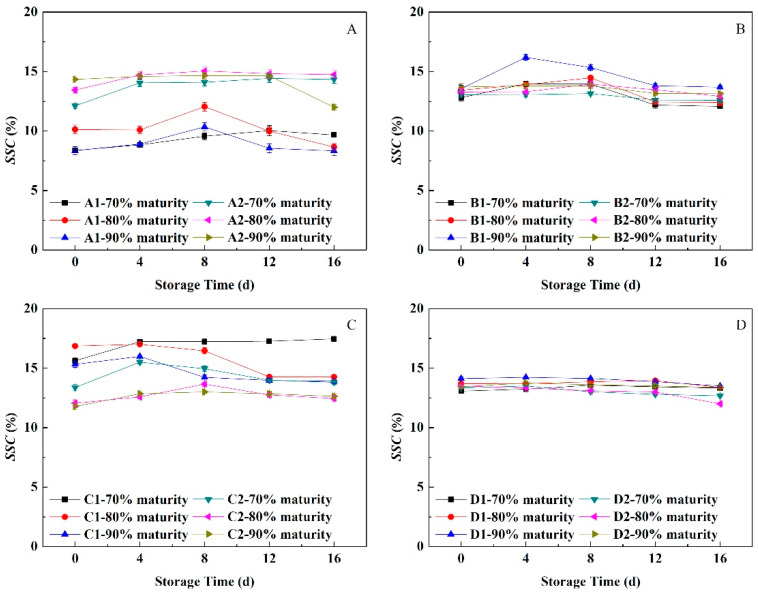
Changes in the soluble solids contents of peach fruits having different flesh types during NFTS. A1, A2 represent ‘Yuhualu’, ‘Xiahui 5’; B1, B2 represent ‘Xiahui 6’, ‘Hujingmilu’; C1, C2 represent ‘Xiacui’, ‘Qinwang’; D1, D2 represent ‘frederic’, ‘Babygold 9’. Soft-melting (**A**), hard-melting (**B**), stonyhard (**C**), non-melting (**D**). Each point represents means ± SE of three replicates.

As shown in Figure 8, the *SSC* in fruit at different maturity levels changed little during the NFTS period, with an upward trend in the early stage and a slight decline in the later stage. The *SSC* of soft-melting ‘Yuhualu’ at 80% and 90% maturity peaked 4 days earlier than that of fruit at 70% maturity. The *SSC* of Xiahui 6’ at 90% maturity peaked 4 days earlier than those at 70% and 80% maturity. The *SSC* of ‘Hujingmilu’ did not change much during NFTS, with 70% > 80% > 90% maturity. The *SSC* of ‘Xiacui’ at 70% maturity increased rapidly during the early stage of storage and then tended to be stable. The *SSC*s of fruit at 80% and 90% maturity levels decreased on the 4th day of storage. The *SSC*s of ‘Qinwang’ at 80% and 90% maturity levels peaked 4 days later than at 70% maturity. The *SSC* of non-melting ‘Frederick’ at 80% maturity peaked 4 days later than at 70% maturity, and the *SSC* at 90% maturity increased slightly in the early stage and then declined slowly. The *SSC* values of ‘Babygold 9’ at all three maturity levels declined slowly after peaking on the 4th day of storage. The *SSC* values of peach fruit with different flesh types changed slowly during NFTS and tended to be stable with the extension of the storage time.

#### 3.8.2. Changes in Sugar Content and Sugar-Acid Ratio

**Figure 9:**
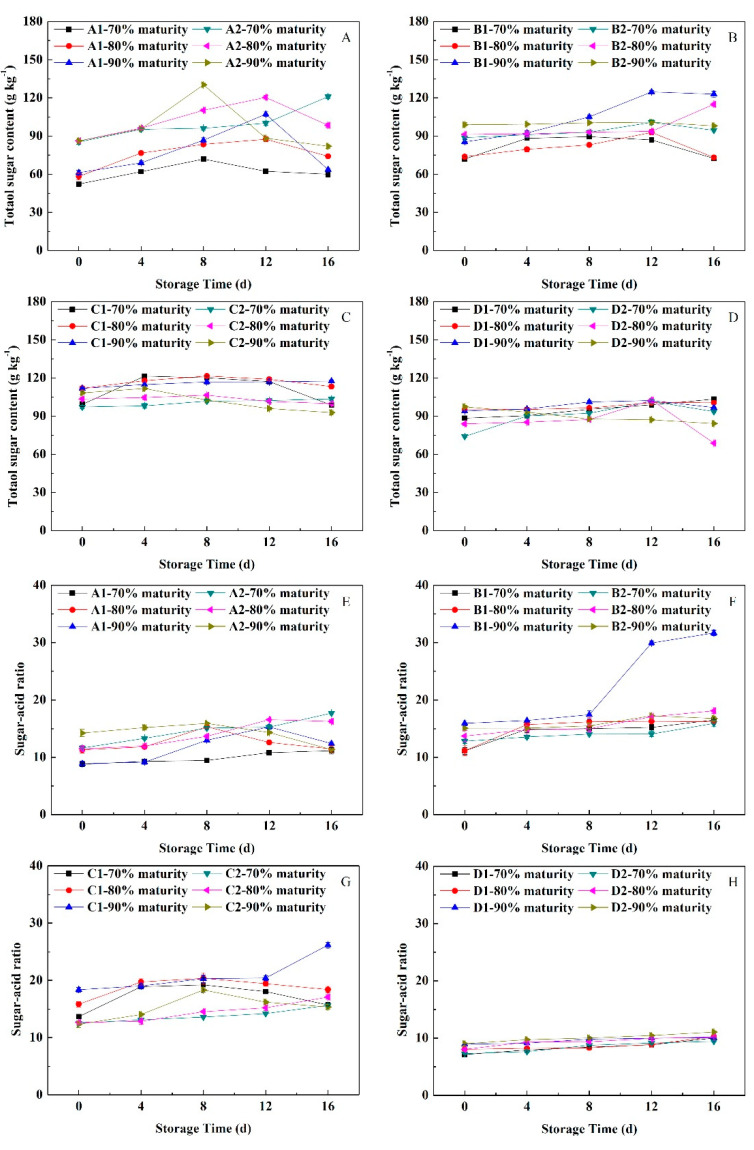
Changes in the sugar contents and sugar-acid ratios of peach fruits having different flesh types during NFTS. A1, A2 represent ‘Yuhualu’, ‘Xiahui 5’; B1, B2 represent ‘Xiahui 6’, ‘Hujingmilu’; C1, C2 represent ‘Xiacui’, ‘Qinwang’; D1, D2 represent ‘frederic’, ‘Babygold 9’. Soft-melting (**A**,**E**), hard-melting (**B**,**F**), stonyhard (**C**,**G**), non-melting (**D**,**H**). Each point represents means ± SE of three replicates.

As shown in Figure 9, the total sugar content of ‘Yuhualu’ at 70% maturity increased gradually in the early storage stage and then decreased slowly. The peak values of fruit at 80% and 90% maturity occurred on the 12th day of storage. The total sugar content of ‘Xiahui 5’ at 70% maturity gradually increased, and the level in fruit at 90% maturity peaked 4 days earlier than those at 80% maturity. The total sugar contents of ‘Xiahui 6’ at the three maturity levels peaked on the 12th day of storage. The content in ‘Hujingmilu’ at 70% maturity continued to increase along with storage time. The total sugar content of ‘Xiacui’ at 70% maturity increased rapidly at the beginning of storage and then decreased slowly, whereas the ranges at the 80% and 90% maturity levels were smaller. The total sugar content of ‘Huayu’ at 90% maturity peaked 4 days earlier than that at 80% maturity, whereas the level at 70% maturity increased slowly. The total sugar contents of non-melting ‘Frederick’ at the three maturity levels increased continuously under NFTS conditions. The total sugar contents of ‘Babygold 9’ at the 70% and 80% maturity levels peaked on the 12th day of storage, whereas the contents at the 90% maturity level decreased slowly.

During NFTS, the changes in the sugar-acid ratios and total sugar contents of peach fruit having different maturity levels were similar, although there were slight differences among the cultivars. The sugar-acid ratio of ‘Yuhualu’ at 80% maturity peaked 4 days earlier than at 90% maturity, and the ratio at 70% maturity increased continuously during storage. The sugar-acid ratios of ‘Qinwang’ at 80% maturity and of ‘Xiahui5’ at 80% and 90% maturity increased continuously. The sugar-acid ratios of ‘Babygold 9’ at the three maturity levels increased slowly during NFTS. Thus, NFTS could effectively delay the decreases in the sugar-acid ratios and total sugar contents of peach fruit.

### 3.9. Acceptability

Significant differences in acceptability were observed among maturity degrees for 8 peach cultivars (Table 3). The acceptability of the 4 flesh-type peach fruit after NFTS for 16 days followed the order 80% > 90% > 70% maturity. After NFTS, the acceptability order of consumers on the same fruit maturity degree was hard-melting > stonyhard > non-melting > soft-melting.

## 4. Discussion

Harvest maturity level and storage temperature are important factors that affect the quality and storage properties of fruit, and selecting the appropriate values is of cardinal significances to improving the storage tolerance and commodity value after storage [19]. Crisosto et al. [20] reported the importance of the stage of maturity regarding fruit commercialization and consumption, and identified firmness as the most appropriate maturity index. A too low or too high harvest maturity level is not conducive to the improvement of fruit storage and shelf-life quality. Late harvesting may accelerate the ripening and senescence processes of fruit, and fruit that is harvested too early may not meet the edible quality requirements after storage [21,22,23]. It’s desirable to keep lower storage temperatures to maintain fruit quality, and is necessary to harvest less ripe fruit to extend storage periods [24,25].

Fruit having a low harvest maturity level are more sensitive to the storage environment, and are prone to cold damage and freezing damage, whereas fruit having a higher maturity level have poor adaptability to the storage environment. In addition, the sensitivity to the environment reduced, and the physiological stability is low [26]. In the current investigation, the mass loss in peach fruit was significantly influenced by the harvest maturity stages and NFT. The mass loss of most of the peach cultivars in NFTS exhibited a significantly decreasing trend from 70% to 90% maturity and a significantly increasing trend during NFTS (Figure 1). The variation in the mass loss at different harvest maturities may be attributed to the differences in respiration rates (Figure 6). In addition, the respiration rate of the fruit decreased as the maturity level increased, whereas the ethylene release rate showed the opposite trend. Similar findings were observed in the other fruits [27,28]. Ding et al. [29] found that in a study of the post-harvest physiology and quality changes of kiwifruit that fruit with a firmness of 11.5 kg cm^−2^ maintain a slower respiration rate at the end of low temperature storage, higher firmness and more stable *SSC* compared with other maturity levels (fruit hardness of 8.5, 12 and 15 kg cm^−2^). In a study of ambarella fruit storage, fruit at the maturity II level (fruit with 75% green skin) during cold storage maintain good quality after 14 days of storage [30]. Here, under NFTS conditions, the water loss of peach with different flesh types accelerated as the maturity level increased, whereas the firmness decreased faster. The values of a* and a*/b*, as well as the Chl and Ant contents, of peels were the greater at the 90% maturity level during storage. The *SSC* and sugar-acid ratio both showed a tendency to increase or decrease slowly during NFTS, but the decline rate of fruit at 90% maturity was faster than those at 70% and 80% maturity. Thus, fruit at 80% maturity, compared with 90% maturity, maintained more fruit water, edible firmness and flavor, and the rate of respiration and ethylene release were relatively moderate, which could effectively slow down the fruit aging process. Therefore, 80% maturity was the optimal maturity for NFTS.

The flesh type of peach fruit influences its storage and transportation quality, and it effects the commodity value [18,31,32]. The performance of fruit quality indicators varied greatly [33]. Yang et al. [34] compared the storage characteristics of peach cultivars having different flesh types (soft-melting, hard-melting and non-melting) at normal temperature and found that hard-melting peach had a better storage effect during the shelf life. Karakurt et al. [31] reported that non-melting peach fruits had significantly higher skin lightness and hue values, firmness, soluble solids and total carotenes and xanthophylls contents than melting fruits. Here, we studied the changes in the fruit quality indexes of four different peach flesh types under NFTS conditions and found that the firmness of soft-melting peach decreased fastest. Additionally, the respiration rate was higher than those of the other flesh types. The ethylene release rate was faster, and the overall fruit quality after 16 days of NFTS was worse than those of the other flesh types. The non-melting peach had a slower water loss rate than those of other flesh types, which was greatly related to the characteristics of non-melting peach, such as dense flesh and less juice [35]. The *SSC* and total sugar content of hard-melting and stonyhard peach were higher than those of the other flesh types during NFTS. Both fruit types maintained high firmness levels at the end of storage. The respiration intensity and the amount of ethylene released were similar, but in terms of fruit coloring, the hard-melting peach performed better than the stonyhard peach.

## 5. Conclusions

Compared with soft-melting and non-melting peach fruit, hard-melting and stonyhard fruit had a higher soluble solids content and total sugar content, lower respiratory intensity and less ethylene production. Additionally, hard-melting peach fruit had a better fruit coloring performance than stonyhard fruit during storage. Thus, hard-melting peach fruit maintained a greater commercial value and better storage characteristics than other flesh-type peach, and the 80% maturity level was optimal for near-freezing point temperature storage. In the near-freezing point temperature storage of peach fruits of different flesh-types, it is very important to maintain the commodity quality and prolong the shelf life of peach fruits by judging the maturity scientifically and evaluating the changes of fruit appearance quality and internal quality.

## Figures and Tables

**Table 1:** The index of absorbance difference of peach fruit having different flesh types at different maturity levels.

Fleshy Type	Cultivar	70% Maturity	80% Maturity	90% Maturity
Soft-melting	‘Yuhualu’	1.01 ^a^	0.90 ^b^	0.33 ^c^
	‘Xiahui 5’	1.15 ^a^	0.45 ^b^	0.35 ^c^
Hard-melting	‘Xiahui 6’	1.07 ^a^	0.81 ^b^	0.74 ^c^
	‘Hujingmilu’	0.87 ^a^	0.43 ^b^	0.18 ^c^
Stonyhard	‘Xiacui’	0.72 ^a^	0.42 ^b^	0.30 ^c^
	‘Qinwang’	0.46 ^a^	0.32 ^b^	0.27 ^c^
Non-melting	‘Frederick’	1.27 ^a^	0.60 ^b^	0.29 ^c^
	‘Babygold 9’	1.50 ^a^	0.81 ^b^	0.30 ^c^

Different superscript letters in the same row show significant statistical difference (*p* < 0.05).

**Table 2:** Changes in the freezing point temperatures of peach fruit having different flesh types at different maturity levels.

Fleshy Type	Cultivar	Freezing Point (°C)					
		70% Maturity		80% Maturity		90% Maturity	
		Mean	Range	Mean	Range	Mean	Range
Soft-melting	‘Yuhualu’	−0.12	−0.3~0	−0.2	−0.4~0	0.14	−0.1~0.3
	‘Xiahui 5’	−0.01	−0.1~0.1	−0.08	−0.3~0.2	0.12	0~0.3
Hard-melting	‘Xiahui 6’	0.03	−0.2~0.3	−0.09	−0.2~0.1	0.03	−0.3~0.2
	‘Hujingmilu’	−0.52	−0.7~−0.4	−0.31	−0.6~−0.1	−0.48	−0.7~−0.2
Stonyhard	‘Xiacui’	−0.47	−0.9~−0.2	−0.39	−0.7~−0.2	−0.24	−0.5~0
	‘Qinwang’	−0.85	−1.2~−0.5	−0.57	−0.7~−0.5	−0.68	−0.8~−0.6
Non-melting	‘Frederick’	−0.31	−0.8~−0.1	−0.53	−0.8~0	−0.65	−0.9~−0.3
	‘Babygold 9’	−0.8	−0.9~−0.7	−0.64	−0.8~−0.5	−0.9	−1.2~−0.6

**Table 3:** Acceptability of 4 flesh-type peach with different maturity degrees after near-freezing temperature storage for 16 days.

Fleshy Type	Cultivar	70% Maturity	80% Maturity	90% Maturity
Soft-melting	‘Yuhualu’	5.28 ^c^	7.21 ^a^	6.02 ^b^
	‘Xiahui 5’	5.63 ^c^	7.39 ^a^	6.26 ^b^
Hard-melting	‘Xiahui 6’	6.45 ^c^	9.23 ^a^	8.26 ^b^
	‘Hujingmilu’	6.73 ^c^	9.15 ^a^	8.04 ^b^
Stonyhard	‘Xiacui’	6.16 ^c^	8.32 ^a^	7.25 ^b^
	‘Qinwang’	6.27 ^c^	8.25 ^a^	7.07 ^b^
Non-melting	‘Frederick’	6.03 ^c^	7.84 ^a^	6.57 ^b^
	‘Babygold 9’	5.78 ^c^	7.81 ^a^	6.34 ^b^

Different superscript letters in the same row show significant statistical difference (*p* < 0.05).

## Data Availability

The data presented in this study are available on request from the corresponding author.
